# Human Fertility, Molecular Genetics, and Natural Selection in Modern Societies

**DOI:** 10.1371/journal.pone.0126821

**Published:** 2015-06-03

**Authors:** Felix C. Tropf, Gert Stulp, Nicola Barban, Peter M. Visscher, Jian Yang, Harold Snieder, Melinda C. Mills

**Affiliations:** 1 Department of Sociology/ ICS, University of Groningen, Groningen, Netherlands; 2 Department of Population Health, London School of Hygiene and Tropical Medicine, London, England; 3 Department of Sociology/Nuffield College, University of Oxford, Oxford, England; 4 The Queensland Brain Institute, University of Queensland, Brisbane, Australia; 5 The University of Queensland Diamantina Institute, The Translational Research Institute, Brisbane, Australia; 6 Department of Epidemiology, University of Groningen, University Medical Center Groningen, Groningen, Netherlands; Institute of Metabolic Science, UNITED KINGDOM

## Abstract

Research on genetic influences on human fertility outcomes such as number of children ever born (NEB) or the age at first childbirth (AFB) has been solely based on twin and family-designs that suffer from problematic assumptions and practical limitations. The current study exploits recent advances in the field of molecular genetics by applying the genomic-relationship-matrix based restricted maximum likelihood (GREML) methods to quantify for the first time the extent to which common genetic variants influence the NEB and the AFB of women. Using data from the UK and the Netherlands (N = 6,758), results show significant additive genetic effects on both traits explaining 10% (SE = 5) of the variance in the NEB and 15% (SE = 4) in the AFB. We further find a significant negative genetic correlation between AFB and NEB in the pooled sample of –0.62 (SE = 0.27, p-value = 0.02). This finding implies that individuals with genetic predispositions for an earlier AFB had a reproductive advantage and that natural selection operated not only in historical, but also in contemporary populations. The observed postponement in the AFB across the past century in Europe contrasts with these findings, suggesting an evolutionary override by environmental effects and underscoring that evolutionary predictions in modern human societies are not straight forward. It emphasizes the necessity for an integrative research design from the fields of genetics and social sciences in order to understand and predict fertility outcomes. Finally, our results suggest that we may be able to find genetic variants associated with human fertility when conducting GWAS-meta analyses with sufficient sample size.

## Introduction

Recent research within both biology [1–4] and demography [1,5,6] demonstrates a genetic component of human fertility, namely the number of children ever born (NEB) and the age at first birth (AFB) of women, explaining up to 40–50 percent of the observed, respectively phenotypic variance in these traits. The well-established negative relationship of late AFB with lower NEB [7,8] appears to be partly genetic, suggesting that natural selection favored a younger age at first birth over the Twentieth century [2–4]. Genetic studies examining the relationship between NEB and AFB, however, have been solely based on twin [2,9] or other family designs [3,4] that use data on expected genetic differences among relatives to estimate the genetic component underlying these traits. Although these studies pervade in behavioral genetics, they can only draw indirect inferences about genetic contributions and suffer from problematic assumptions and practical limitations (critical discussions on, for example, the equal environment assumption (EEA) can be found in ref [10–12]). This approach is limited for further reasons. First, by virtue of their design, twin studies inherently require pairs of siblings and therefore exclude individuals from low fertility families, particularly only children, which may be problematic for the generalization of results. Second, dizygotic twinning is in contrast to monozygotic twinning genetically based [13,14], which means that dizygotic twins potentially carry genes important for high fertility. Therefore, the use of monozygotic and dizygotic twins to investigate fertility questions in the classic twin design leads to a non-random genetic stratification and might bias variance estimates. Finally, a practical limitation of family designs is that they require data from multiple family-members, which are obviously more difficult to gather than data on unrelated individuals.

An ideal design to examine the genetics of fertility would be a direct estimate using single nucleotide polymorphisms (SNPs) across the entire genome for unrelated individuals who do not share the same micro environment, which was first applied to height as a model complex trait [15,16]. This type of data and the corresponding statistical tools for genome-wide complex trait analyses (GCTA, see ref [17]) have recently become available and are already well-established in the fields of genetic epidemiology [18], psychology [19,20] and sociogenetics [21,22].

The current study exploits recent advances in the field of molecular and quantitative genetics by applying genomic-relationship-matrix restricted maximum likelihood (GREML) methods to quantify for the first time the extent to which common genetic variants influence both the NEB and the AFB of women. We applied both uni- and bivariate models to these traits producing unbiased estimates of their common SNP heritability and the extent to which the association between earlier AFB and higher lifetime fertility (NEB) is due to a (negative) genetic correlation between AFB and NEB [23]. This not only helps us to understand the relationship between the AFB and NEB, but also allows an assessment of whether genes are associated with a reproductive advantage, indicating natural selection in contemporary, industrialized populations.

In contrast to twin and family designs, the GREML approach is free of confounding from shared environmental effects between close relatives because the method can be applied in a sample of unrelated individuals [15,16]. The GREML analyses make use of the genetic similarity between pairwise unrelated individuals as captured by all common SNPs and correlate the genetic similarity with the phenotypic similarity between individuals (see material and methods). To ensure accurate and well-powered estimates, particularly for the bivariate model [24], we pooled data sources to estimate the genetic influence on all outcomes of interest (see material and methods). We utilize two large cohorts, one from the Netherlands (NL, N = 4,338) and one from the United Kingdom (UK, N = 2,420, for descriptive statistics see Table 1). In both populations, resemblance in fertility outcomes has been reported for relatives [25–27] using intergenerational comparisons with survey data. However, no distinction between genetic and environmental effects responsible for this pattern could be made so far. After quality control of the merged genetic data files, we used more than 1 million SNPs to estimate the genetic relationships among the individuals (see material and methods) and subsequently the genetic variance components.

**Table 1:** Descriptive statistics of the female TwinsUK and Lifelines samples.

	*TwinsUK*				*LifeLines*			
	Mean	SD	Min-Max	N	Mean	SD	Min-Max	N
Birth year	1951	13	1919–1987	2420	1960	11	1920–1989	4338
AFB	25.70	4.74	15–44	1951	26.83	4.26	16–43	4016
NEB	2.07	1.21	0–9	1990	2.25	1.20	0–9	2875

Note that the N for the age at first birth (AFB) is different from the N for number of children ever born (NEB). The reason for this is that only women older than 45 have been included in the analysis of NEB. For example, a 35 years old woman with a first child is part of the analysis for AFB but not for NEB. Therefore in the Lifelines cohorts the N for AFB is larger than for NEB, because it contains a large proportion of women younger than 45. This also implies that the average AFB in the more recent birth cohorts is younger than in the overall population.

The most successful and popular design to detect the approximate location of genetic variants associated with a complex trait is the meta-analyses of genome-wide association studies (GWAS) from multiple samples. In lieu of this, our assessment of the genetic effects of common SNPs based on the pooled samples shape the expectations to find individual variants when conducting a GWAS. We account for population stratification effects by adjusting for the first 20 principal components in our GREML models. Population stratification refers to allele frequency differences due to systematic ancestry differences. Population stratification can cause spurious associations if not adjusted properly (for additional information see [28]). We furthermore correct for country and birth cohort effects as well as dizygotic twinning. From the twin data only singletons are included, so that close relatives do not contribute to the estimates.

This study has several important implications for research in demography, genetics and biology. We know surprisingly little about genetic effects on human fertility on a population level, yet it is crucial for our understanding of fertility, the interpretation of related social science research in this field [21,22,29–31], and broader questions of modern human evolution [3,4,32,33]. We first discuss the importance of adopting an integrative multidisciplinary approach to understand human fertility before proceeding with an introduction of the methods and the presentation and discussion of our findings.

### Towards an integrative approach in human fertility research

The term ‘fertility’ takes on different meanings in demography, reproductive medicine and biology [7]. In demography, fertility refers to performance, specifically the two interrelated aspects of the tempo of childbearing (in our case age at first childbirth, AFB) and the quantum or number of children ever born (NEB) in a certain period [34]. In reproductive medicine, fertility defines the ability/inability of couples to conceive and have children given unprotected intercourse [35]. In biology, AFB and NEB have become central indicators for individual fitness as the successful transmission of genes to the next generation in post-industrial societies [4,33], with particularly NEB shown to be nearly perfectly correlation with alternative measures [2,36]. Due to improvements in hygiene and the reduction in prenatal, infant and child mortality in industrialized societies, NEB has emerged as the gold standard to measure lifetime reproductive success indicating biological fitness [33].

In the last decades, industrialized societies have experienced massive changes in both the postponement of AFB and drop in the total number of offspring, which cannot mainly be attributed to genetic or biological factors [7,37]. Rather, human reproduction is influenced by three analytically distinct but empirically interrelated factors: 1) genetic and biological fecundity (i.e., length of reproductive period, infertility diseases), 2) the environment (i.e., institutional and family structures); and, 3) reproductive choice of individuals (i.e., planned behavior, latent individual and partner characteristics).

Previous research has successfully demonstrated that there is a genetic component to reproduction with over 70 genome-wide association studies (GWAS) published for 32 traits and diseases associated with reproduction found in [14]. This includes identification of genes such as those related to age at menarche [38,39] and menopause [40–43], and endometriosis [44]. Environmental factors, such as women’s gains in education and labor market participation, gender equity and economic uncertainty have been demonstrated to strongly impact the tempo and quantum of fertility (for reviews see ref [7,37]). Studies of reproductive choice have examined the predictive power of fertility intentions on behavior and often position reproductive choice in a socio-psychological framework that consists of attitudes (perceived costs and benefits), norms (influence social network) and perception of control over individual choice [45,46].

A bivariate twin model in a study by Rodgers and colleagues [47] suggests an interrelation between reproductive choice and genetic factors, providing evidence for shared genetic effects on the decision to have a first child and the number of children during lifetime. It is therefore likely that biological fecundity, the environment and reproductive choice not only interact with each other, but that genes also influence reproductive choice. Genetic endowment in social science fertility research has been virtually ignored [37], yet may be of major importance when drawing conclusions about observable associations.

If the quantum of fertility in the form of NEB is at least partly genetically influenced, this implies that certain SNPs have a higher chance to be successfully transmitted to the next generation than others, and by extension that the allele frequency might change due to natural selection, indicating evolution. If the negative relationship between AFB and NEB is partly genetic, this would indicate that the AFB was under natural selection during the Twentieth century and that more recent birth cohorts may carry a higher genetic predisposition for an earlier AFB.

Using a family-design, findings from the Framingham Heart Study demonstrated that the same genes influencing NEB are negatively correlated with the AFB [4]. The authors subsequently predict that selective changes in the disposition for the timing of the first child predict the decrease in the AFB for subsequent generations. The study design, however, is based on correlations between relatives and the estimates can therefore be inflated by shared environmental factors such as family norms that are important for fertility [48]. Family designs cannot robustly discriminate between the case that the correlation between NEB and AFB is environmentally caused, and natural selection, in which case the correlation is genetically caused and the allele frequencies of the genome might change [33]. This limitation leaves a less desirable practical solution “…to note the issue and remain modest in drawing conclusions” ([33] p. 614). In the current study, our design does make it possible to directly draw conclusions about modern natural selection based on information derived from the field of molecular genetics. When the trait of interest, here the age at first birth, does not genetically covary with fertility, a genetic response to selection will not occur [49].

## Material and Methods

### Ethics Statement

Written informed consent has been given by each TwinsUK and Lifelines participant. This research was approved by the Department of Sociology’s Departmental Research Ethics Review Committee both at the University of Groningen and the University of Oxford. All data had been anonymized before we received it.

### Samples

For the Netherlands, we use data from the LifeLines cohort study, a multi-disciplinary prospective population-based cohort study examining in a unique three-generation design the health and health-related behaviours of 167,729 persons living in the North of The Netherlandsincluding genotype information from more than 13,000 unrelated individuals [50]. It employs a broad range of investigative procedures in assessing the biomedical, socio-demographic, behavioural, physical and psychological factors which contribute to the health and disease of the general population, with a special focus on multi-morbidity and complex genetics.

For the UK, we use data from TwinsUK, the largest adult twin registry in the country with more than 12,000 respondents [50]. Due to our analytical strategy, we randomly selected only one twin for analysis and controlled for dizygotic twinning as a genetically related process. We recognize that for generalizability a population-based sample such as LifeLines is more desirable for the models we present. The descriptive statistics of the phenotypic variables in the genotyped subsamples with full fertility information are shown in Table 1.

### Genotypes

Since genotyping had been performed using different chips in the UK and the Netherlands, we use imputed data to aid the alignment of both datasets. The HapMap3 imputation panel has been shown to be reliable for GREML analysis [26].

We received genotype data from TwinsUK and Lifelines, which we imputed according to the 1000 genome panel after which we selected HapMap3 SNPs with an imputation score larger than 0.6. For quality control (QC), we excluded the SNPs with a larger missing rate than 3%, lower minor allele frequency than 1% and which failed the Hardy-Weinberg equilibrium test for a threshold of 10^−6^ for both datasets. We merged the TwinsUK and the Lifelines samples and quality controlled the merged dataset in the same way again. On average 1,017,420 SNPs could be utilized to estimate the GRM between individuals. We used the software Plink [51] for the quality control and merging of the two dataset.

### Phenotypes

#### Number of children ever born

Number of children ever born measures the number of children a woman has given birth to including stillbirths. This has been asked directly in the twinsUK (“How many children have you given birth to?”) or we constructed it using questions about the year of childbirth of each child. In Lifelines, respondents have been asked to list the birth and death date of children from their current and previous partner with up to 6 children in both categories. For the Lifelines and part of the TwinsUK questionnaires information for the date of death of the children was given. In both datasets less than 0.2% of the children had not reached reproductive age and the correlation of number of children ever born and number of children reaching reproductive age was >0.98.

Since fertility is strongly age dependent, we focus on women with completed fertility history in reference to the phenotype. In general the end of the woman’s reproductive lifespan occurs around the age of 45 [52], thus, we only included women aged 45 or older in our analysis of NEB. Furthermore, in vitro fertilization (IVF)—often related to twinning and multiple births—can bias results if IVF compensates genetically based infertility. However, in our TwinsUK sample, only 60 women reported using IVF who we did not include in the final analyses.

#### Age at first birth

To calculate the AFB, we used information on the year of childbirth of the first child and year of birth of the mother. In TwinsUK, information from an additional behavioral questionnaire directly asking for the age at first birth in 2005 was available. Childless individuals have been set to missing in the analysis.

#### Heritability estimates

The genetic component underlying a trait is commonly quantified in terms of heritability (*h* ^2^) as the proportion of the genetically caused variance $\left(\sigma_{G}^{2}\right)$ over the overall phenotypic variance of the trait (phenotype, *V*_*P*_)) [9]:

$$h^{2}=\frac{\left(\sigma_{G}^{2}\right)}{\left(\sigma_{P}^{2}\right)}$$

Whereas the phenotypic variance is the sum of genetic and environmental $\sigma_{e}^{2}$ variance components.

$$\sigma_{P}^{2}=\sigma_{G}^{2}+\sigma_{e}^{2}$$

The methods we applied have been detailed elsewhere [15–17]. Briefly, we applied a mixed linear model

y=g+e

where y is an Nx1 is vector of dependent variables, N is the sample size, g is the Nx1 vector with each of its elements being the total genetic effect of all SNPs for an individual, and e is an Nx1 vector of residuals.

We have g~ $N(0,\sigma_{G}^{2}A)$ and e~ $N(0,\sigma_{e}^{2}I)$, where $\sigma_{G}^{2}$ is the genetic variance by all SNPs, ***A*** is the genetic relationship matrix (GRM) estimated from SNPs, $\sigma_{e}^{2}$ is the residual variance and ***I*** is an identity matrix. The variance components are estimated using the restricted maximum likelihood (REML) approach. The NEB is not normally distributed (see S1 Fig). This might bias the inference, whereas simulation studies show that there is no bias even for binary traits [18]. Still, we base our p-values on likelihood-ratio tests, comparing the full model with one contraining genetic effects to be zero [53].

The estimates of heritability obtained using GREML can be interpreted as the proportion of variance of a trait based on a large set of common genetic variants genotyped. The method is based on the genetic relatedness among individuals measured on about one million of SNPs.

This analysis has been extended to a bivariate approach by Lee and colleagues [23] to estimate unbiased genetic correlation based on a standard bivariate linear mixed model combined with the genome-wide genetic relatedness matrix.

#### Genetic correlation

The genetic correlation (*r*(*G*)) is an estimate that standardizes the genetic covariance between two traits *Cov*(*G*_*t*1,*t*2_) by the genetic variance of both traits:

$$r(G_{t1,t2})=\frac{Cov(G_{t1,t2})}{\sqrt{V_{G_{t1}}}*\sqrt{V_{G_{t2}}}}$$

If the genetic correlation between two traits is 1, all genetic variance in trait 1 and 2 has a common base. If the genetic correlation is 0, the genetically based variance between trait 1 and 2 are independent.

#### Phenotypic and genetic correlation analysis

The phenotypic correlation between two traits *r*(*P*_*t*1,*t*2_) is the sum of genetic and environmental influences shared across traits and can be estimated like this:

$$r(P)=\sqrt{h_{t1}{}^{2}}*r(G_{t1,t2})*\sqrt{h_{t2}{}^{2}}+\sqrt{e_{t1}{}^{2}}*r(E_{t1,t2})*\sqrt{e_{t2}{}^{2}}$$

whereas $h_{ti}^{2}$ is the heritability of trait i in the model and $e_{ti}^{2}$ is the environmental or residual variance contribution for the trait, standardized for the overall variance

$$e^{2}=\frac{\sigma_{e}^{2}}{\sigma_{P}^{2}}=1-h^{2}$$

and *r*(*E*_*t*1,*t*2_) is the environmental or residual correlation between the traits (for the estimates of environmental effects see S3 Table. We can solve this to compute the fraction of the phenotypic correlation explained by the genes (or the environment respectively the residuals). For the transformation of standard errors, the delta-method has been applied [54].

## Results

Table 1 shows the descriptive statistics for both traits in the TwinsUK and the Lifelines cohorts. Overall the AFB is around one year later in the Dutch (26.83) than in the UK cohort (25.70) and the UK women are about 9 years older. These characteristics are interrelated, since Europe experienced a massive postponement in the AFB during the second half of the Twenties century [7], so the larger proportion of younger individuals leads to a later average AFB in the data.

To combine the cohorts, both fertility measures, AFB and NEB, have been standardized by country (Z-transformation) and the NEB has been log transformed to approach normal distribution (see S1 Fig for distributions and S1 Table for the model estimation of all alternative transformations—estimates are robust across transformations).

### The correlation between AFB and NEB

**Fig 1:**
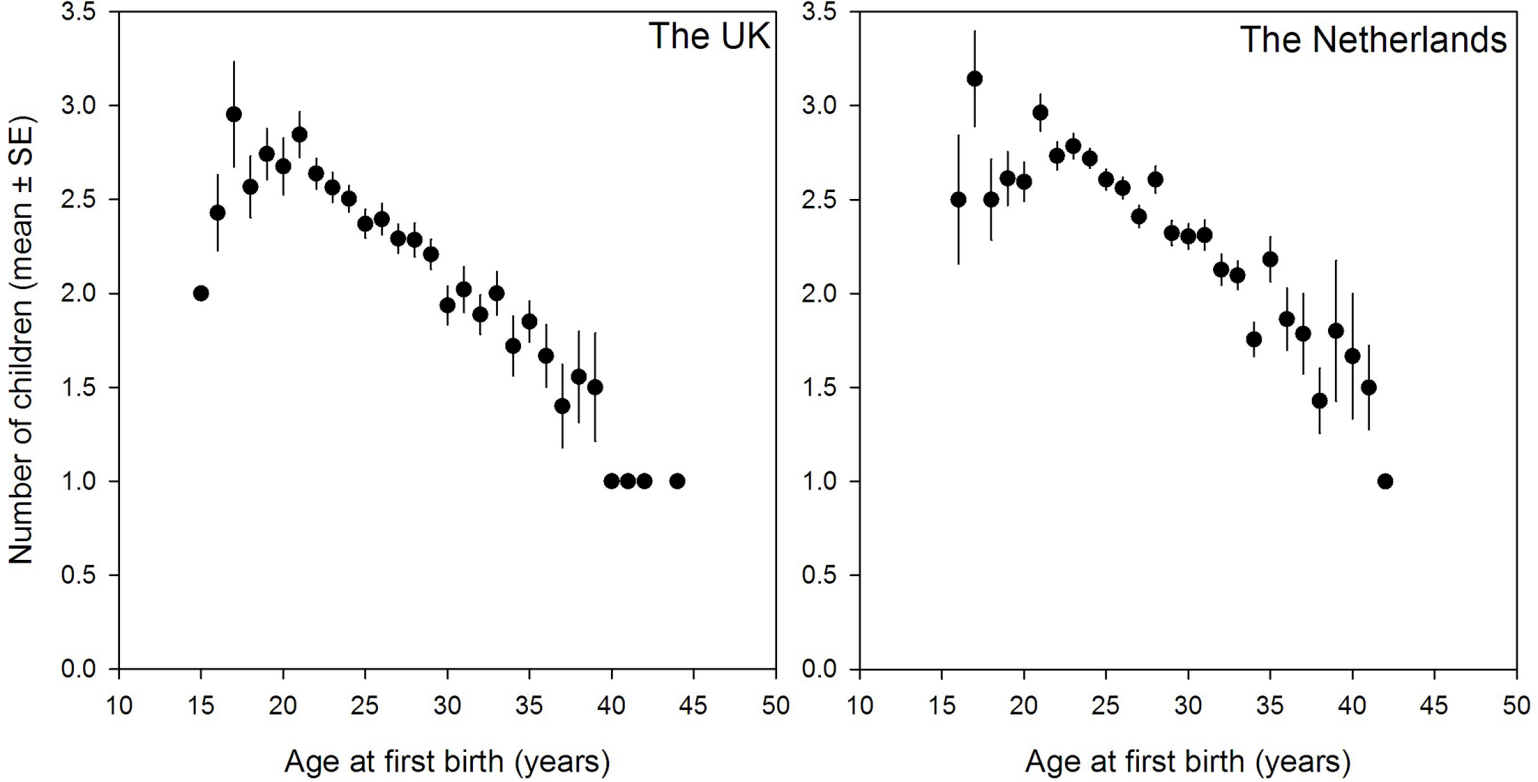
The association between age at first birth and number of children ever born in the British and the Dutch cohorts.

In line with previous studies, women who had their first child at a later age had a lower number of children ever born (Fig 1) in both the British and Dutch sample. The observable correlation for individuals with full information on both traits (therefore excluding all childless individuals, individuals younger than 45 and individuals without information about the AFB) between AFB and NEB is -0.32 (N = 1,521) in the UK cohorts, -0.26 (N = 2,553) in the Dutch cohorts and -0.28 (N = 4,074) for the standardized measures in the pooled cohorts (-0.27 if estimated from the residuals of all covariates, not listed).

### SNP heritability of AFB and NEB

Table 2 depicts the SNP based heritability (h^2^_SNP_) estimated from the univariate models for AFB and NEB. Both traits have a significant genetic component, with h^2^_SNP_ for NEB of 0.10 (SE 0.05) and for the AFB of 0.15 (SE 0.04). These results suggest that additive effects of common SNPs explain 10% of the variance in the NEB and 15% of the variance in the AFB of women.

**Table 2:** Heritability estimates of NEB and AFB for the pooled sample of women from the UK and the Netherlands using information from about 1 million SNPs.

	h^2^_SNP_ (SE)	*p-value* ^c^	N
Number of children ever born^a^	0.10 (0.05)	0.02	4865
Age at first birth^b^	0.15 (0.04)	0.0004	5967^d^

a: standardized by country and log transformed to adapt the distribution

b: standardized by country

c: p-values are based on likelihood-ratio tests, the reference model constraints genetic effects to be 0; Please find estimates of untransformed variables in S2 Table

d: The N for age at first birth is larger than for number of children ever born. The reason is that only women with completed fertility history are included for the latter (for discussion see material and methods and Table 1.).

### Bivariate GREML analysis of AFB and NEB

Table 3 shows the results for the bivariate GREML model of AFB and NEB, including the genetic correlation between both traits. The genetic correlation would be—1.00/1.00 if all genetic effects of AFB and NEB are shared and 0 if the genetic effects of AFB and NEB would be completely independent. The genetic correlation estimate is -0.62 (SE 0.27) and significantly different from 0 (p-value = 0.02), meaning that genes that lead to a later age at AFB are indeed negatively associated with the NEB. Based on these estimates, genetic effects lead to a phenotypic correlation of -0.07 (0.03) between AFB and NEB, whereas the overall correlation estimated from the fitted model is -0.38 (SE = 0.02). Therefore around 20% of the phenotypic correlation is associated with shared genetic effects across the traits while still the main part is associated with common environmental/residual effects of the AFB and the NEB. The phenotypic correlation estimated from the genetic model is larger than the observed correlation because the bivariate GREML analysis does not require both traits measured on exactly the same set of individuals so that it makes use of additional information, e.g. childless individual for the estimates of NEB. If we only include individuals with full information on both traits in the genetic model—as we do when computing the phenotypic correlation directly—the phenotypic correlation estimated based on the genetic model (-0.29 SE = 0.02) is not significantly different from the observed value based on Pearson correlation (-0.27) and the component due to genetic effects estimated from the GREML model (-0.08 SE = 0.05) is not significantly different from that using all available information (-0.07 SE = 0.03), whereas the inference would be weaker (see S3 Table for the model excluding all individuals with missing information).

**Table 3:** Estimates of the bivariate genetic model for NEB and AFB for the pooled sample of women from the UK and the Netherlands using information from about 1 million SNPs.

h^2^_SNP NEB_ (SE)	h^2^_SNP AFB_ (SE)	r(G)_SNP AFB-NEB_ (SE)	p-value^a^	Phenotypic correlation		N_AFB/NEB_
				Overall (SE^b^)	Due to genetic effects (SE^b^)	
0.08 (0.05)	0.15 (0.04)	-0.62 (0.27)	0.02	-0.38 (0.02)	-0.07 (0.03)	5967/4865 ^c^

NEB: standardized by country and log transformed to adapt the distribution; AFB: standardized by country

a: p-values are based on likelihood-ratio tests, the reference model constraints genetic effects to be 0;—one-tailed (default in GCTA)

b. Standard errors have been transformed using the delta method([54])

c: The N of age at first birth is larger than for number of children ever born. The reason is that only women with completed fertility history are included for the latter (for discussion see material and methods and Table 1). For the full model, including environmental/residual effects see S2 Table.

## Discussion

Using recently developed analytical techniques from molecular genetics we provide direct evidence for a genetic component underlying the AFB and NEB of women in the UK and the NL born during the Twentieth century. Moreover, genetic effects on the tempo (AFB) and quantum (NEB) of human reproduction co-vary, which partly explains why women who start reproducing at an earlier age, have higher fertility.

This genetic association between AFB and NEB can have different origins. Both traits might simultaneously be influenced by the same genetic effects (pleiotropy) or genetic effects on the NEB could be mediated via the AFB—as well as a combination of both. To further examine the causal relations between these factors, actual measured genotypes important for these traits might be integrated in the statistical models [55] in applications such as Mendelian randomization [56]—although this has been challenged [57]. Whatever the underlying cause of the genetic association between NEB and AFB is, as the consequence of this genetic association we expect natural selection acting in modern, industrialized societies, implying that women born in more recent cohorts may be genetically inclined to have an earlier AFB. This prediction of a decrease in AFB, however, is somewhat of a ‘population paradox’ because it strongly contradicts observed fertility trends over the last 50 years. Instead, there has been a massive postponement in the AFB of an average of 4–5 years in nearly all European countries and the US since the 1970s [7].

Although our results seem to raise a paradox, they are well in line with studies on natural fertility populations, such as from Milot and colleagues [3] who observed a decrease in AFB as a response to natural selection in a contemporary population. One probable explanation is that natural selection works in addition to environmental forces and in the opposite direction; with the latter being stronger. Natural fertility populations are assumed to have set fertility norms to maximize reproductive success. With the absence of contraption, the full reproductive potential can be expressed [3]. In European and many industrialized societies, in contrast, environmental changes across the past century such as the use of contraceptives and women’s educational expansion and entry into the labor market have had a strong impact on fertility behavior [7,8,48]. This which has led to a postponement in the AFB even though more recent populations in the Netherlands and the UK are genetically predisposed to an earlier AFB. In that sense, the environment has achieved an evolutionary override.

The discrepancy between observed changes and those predicted by evolutionary processes show parallels to the case of human height. Although natural selection has disinclination for taller individuals at least in US populations [4,58], people still, on average, grow up to be taller than their parents [59]. This is also largely attributed to environmental factors, such as better nutrition and improved health care [60]. Natural selection, however, may also work as tandem with environmental factors: a recent study suggests that, in the Netherlands, natural selection favored taller heights, and thus reinforced the effects of improved environmental quality over the last 150 years [61].

A second potential—and largely interrelated—explanation for the fact that AFB is postponed despite selection towards genes favoring earlier birth is that genes and the environment interact across birth cohorts. Previous twin studies have in fact shown differences in heritability estimates across cohorts and environments in both NEB [6] and AFB [2,31,62,63]. Therefore, independent of additive environmental effects leading to postponement in the AFB, genetic variants important for the AFB may differ across cohorts and populations, so that large changes due to natural selection are not necessarily implied.

The genetic effects estimated in this study represent narrow sense heritability estimated from SNP data. As can be expected [64], they are lower than the estimates of narrow sense heritability (~0.20–0.30) obtained from family designs. Potential reasons for this are, on one hand, the inflation of estimates by shared environmental factors in family designs, but on the other hand true genetic effects of variants that are not captured through linkage disequilibrium with SNPs used in GREML analysis. In order to engage in a more rigorous examination of genetic effects as well as gene-environment interplay, replication in larger datasets and across populations is required. The provision of data with genetic and environmental information continues to grow, as do more advanced analysis techniques [65]. Nonetheless, it becomes obvious that human fertility is both a genuinely biological process as well as a social undertaking. We conclude from our findings that an integrative approach between the social and biological sciences is necessary to better understand the changing patterns in, or even predict future levels of human fertility.

Despite the significant advances in the estimation techniques and sample size of this study, there are two limitations that need to be made explicit. First, the interpretation of NEB in an evolutionary manner implies an interpretation of NEB as a *measure of fitness*. It would be better to have information on the number of children who entered reproductive age or even more appropriate, the number of grandchildren entering reproductive age to have a more precise measure of how far genes have been successfully transmitted across generations. However, the NEB has been shown to be a good measure of reproductive success (see also [33]) due to diminishing mortality during the reproductive lifespan. Recent genetically-informed research furthermore demonstrates that the same genes important for the NEB also influence the number of grandchildren born and therefore have a long-term effects [32]. Second, as opposed to common research in demography [66], it is still uncommon to deal with right censored information (i.e., those who have not yet had a child by the time of observation) in genetic studies. In our case, we have set individuals who remained childless as missings when estimating genetic influences for the AFB, since they did not yet have a child [4,63]. Childless individuals, however, are of great interest for demographic research as well as from an evolutionary perspective—since they are the ones who do not transmit their genes to the next generation. While the structural equation modelling in twin studies provides alternative solutions such as Tobit [67] or ordered models [2] to integrate censored information, there remains no possibility to consider this in current applications of GREML. In general, the observable association can be expected to be stronger when including childless individuals, because all childless women are right-censored cases. However, it has been shown in twin models that it is more difficult to predict the effect on the genetic analysis. Therefore, the extent of empirical differences between our model and survival models must be tackled in future research [67].

To date, thousands of genetic variants have now been successfully linked to physical or psychological traits in the past years [68,69], as well as complex ‘socio-genetic’ traits like educational attainment [29] and also traits related to reproduction [14]. We conclude that our study, based on the same genetic data as in GWAS studies, raises confidence that we will find genetic variants associated with human fertility when conducting GWAS-meta analyses of sufficient sample size.

## Supporting Information

S1 Fig Distributions of the dependent variables. (DOCX)

S1 Table Estimates for NEB and AFB based on both the unstandardized measures and the transformations variables as seen in S1 Table. (DOCX)

S2 Table Genetic (^2^_G_) and environmental variance (^2^_e_) components and covariances (Cov(G), Cov(E)) of the bivariate model for NEB (N = 4865) and AFB (N = 5967) in the pooled sample from the UK and the Netherlands using information from about 1 million SNPs. (DOCX)

S3 Table Estimates of the bivariate genetic model excluding childless individuals and for NEB and AFB for the pooled sample from the UK and the Netherlands using information about 1 million SNPs. (DOCX)
