# Supplementary material for: Human Fertility, Molecular Genetics, and Natural Selection in Modern Societies
Source: PLoS One. 2015 Jun 3;10(6):e0126821. doi: 10.1371/journal.pone.0126821 (PMC4454512; doi:10.1371/journal.pone.0126821)
Supplement: S1 Fig — (DOCX) [file pone.0126821.s001.docx]

**S1 Fig. Distributions of the dependent variables**

The graphs below show for the number of children ever born (S1a Fig.) and the age at first birth (S1b Fig.) the distribution of the measured variables, the distribution of the Z-standardized variables and the log-transformed standardized variables (for the log transformation a constant has been added to the standardized value). S2 Table shows the results for all models on all phenotypes.

S1a Fig. Distribution of number of children ever born across the cohorts


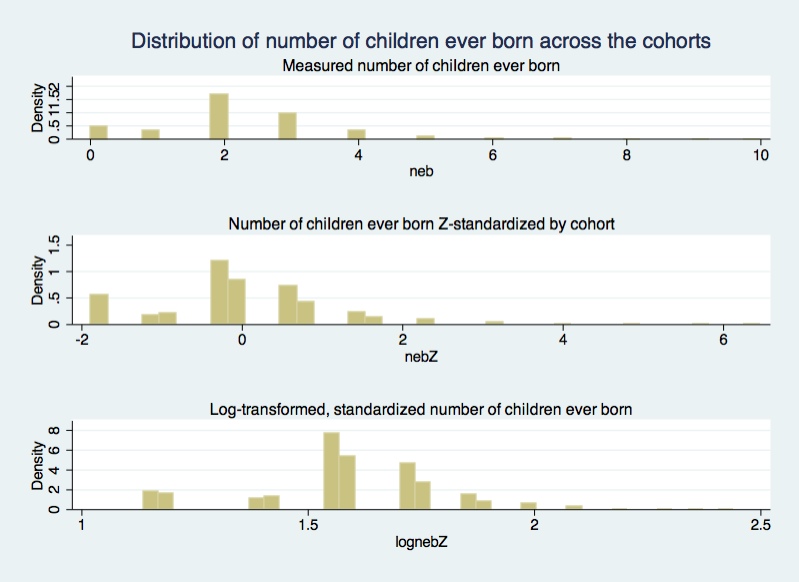


S1b Fig. Distribution of age at first birth across the cohorts
