# Supplementary material for: Human Fertility, Molecular Genetics, and Natural Selection in Modern Societies
Source: PLoS One. 2015 Jun 3;10(6):e0126821. doi: 10.1371/journal.pone.0126821 (PMC4454512; doi:10.1371/journal.pone.0126821)
Supplement: S1 Table — (DOCX) [file pone.0126821.s002.docx]

| **S1 Table. Estimates for NEB and AFB based on both the unstandardized measures and the transformations variables as seen in S1 Fig.** | | | | |
| --- | --- | --- | --- | --- |
|  |  | h^2^_SNPs_ (SE) | *p-value*^a^ | N |
| Number of children ever born | Unstandardized | 0.09 (0.05) | 0.04 | 4865 |
|  | Z-standardized | 0.09 (0.05) | 0.04 |  |
|  | Standardized and log-transformed | 0.10 (0.05) | 0.02 |  |
| Age at first birth | Unstandardized | 0.15 (0.04) | <0.00 | 5967 |
|  | Z-standardized | 0.14 (0.04) | <0.00 |  |
|  | Standardized and log-transformed | 0.14 (0.04) | <0.00 |  |
| a: p-values are based on likelihood-ratio test compare to a reference model in which genetic effects are constrained to be 0 | | | | |
