# Supplementary material for: Human Fertility, Molecular Genetics, and Natural Selection in Modern Societies
Source: PLoS One. 2015 Jun 3;10(6):e0126821. doi: 10.1371/journal.pone.0126821 (PMC4454512; doi:10.1371/journal.pone.0126821)
Supplement: S2 Table — (DOCX) [file pone.0126821.s003.docx]

| **S2 Table. Genetic (**$\sigma$ ^2^_G_**) and environmental variance (**$\sigma$ ^2^_e_**) components and covariances ( Cov(G), Cov(E)) of the bivariate model for NEB (N = 4865) and AFB (N = 5967) in the pooled sample from the UK and the Netherlands using information from about 1 million SNPs** | | |
| --- | --- | --- |
| NEB | $\sigma$ ^2^_G_ (SE) | 0.004 (0.002) |
|  | $\sigma$ ^2^_e_ (SE) | 0.038 (<0.002) |
| AFB | $\sigma$ ^2^_G_(SE) | 0.144 (0.043) |
|  | $\sigma$ ^2^_e_ (SE) | 0.859 (0.044) |
| $\sigma$ ^2^_G NEB, AFB_ (SE) | | -0.014 (0.007) |
| $\sigma$ ^2^_e NEB, AFB_ (SE) | | -0.064 (0.008) |
