# Supplementary material for: Human Fertility, Molecular Genetics, and Natural Selection in Modern Societies
Source: PLoS One. 2015 Jun 3;10(6):e0126821. doi: 10.1371/journal.pone.0126821 (PMC4454512; doi:10.1371/journal.pone.0126821)
Supplement: S3 Table — (DOCX) [file pone.0126821.s004.docx]

| **S3 Table. Estimates of the bivariate genetic model excluding childless individuals and for NEB and AFB for the pooled sample from the UK and the Netherlands using information about 1 million SNPs** | | | | | | |
| --- | --- | --- | --- | --- | --- | --- |
| h^2^_SNPs NEB_ (SE) | h^2^_SNPs AFB_ (SE) | r(G)_SNPs AFB-NEB_ (SE) | p-value^a^ | Phenotypic correlation | | N_AFB/NEB_ |
|  |  |  |  | Overall (SE^b^) | Due to genetic effects (SE^b^) |  |
| 0.09 (0.06) | 0.07 (0.06) | -1.0 (0.63) | 0.06 | -0.29 (0.02) | -0.08 (0.05) | 4072/4072 |
| NEB: standardized by country and log transformed to adapt the distribution; AFB: standardized by country; a: p-values are based on likelihood-ratio tests, the reference model constraints genetic effects to be 0; – one-tailed (default in GCTA); b. Standard errors have been transformed using the delta method(^1^); Note that the estimate of the genetic correlation in S3 Table is lower than in Table 3 of the main text (-0.62 [SE 0.27]). However, the difference is not significant. We conclude that both models support a negative association between NEB and AFB. The worse inference of the model presented in S3 makes it less trustworthy than the one shown in the main text  . | | | | | | |
